# Case series on *Trichomonas vaginalis* infections: impact of proper sample collection and diagnostic stewardship

**DOI:** 10.1099/acmi.0.000698.v4

**Published:** 2024-01-31

**Authors:** Ashwini Agarwal, Abhishek Padhi, Anil Chaudhary, Mayuri Bhise, Kinjal Chauhan, Medhavi Sharma, Ramotra Rohini Krishan, Pankhuri Dubey

**Affiliations:** ^1^​ Department of Microbiology, All India Institute of Medical Sciences, Rajkot, India; ^2^​ Department of Obstetrics and Gynaecology, All India Institute of Medical Sciences, Rajkot, India

**Keywords:** *Trichomonas vaginalis*, diagnostic stewardship, sexually transmitted infections, STIs

## Abstract

This paper elucidates the transformative impact of a strategic shift in diagnostic practices in the detection of *Trichomonas vaginalis*. It explores five cases where the implementation of a specific diagnostic protocol led to effective identification of the infection. In-depth discussions and a comprehensive literature review underline the necessity for precise diagnosis and the paramount importance of diagnostic stewardship in managing sexually transmitted infections.

## Data Summary

No new data, tools, software or code were generated.

## Introduction

Trichomoniasis, caused by the protozoan parasite *Trichomonas vaginalis*, is one of the most common non-viral sexually transmitted infections (STIs) worldwide, affecting millions annually [1]. However, its prevalence may be underestimated due to diagnostic inaccuracies often associated with improper sample collection and laboratory methods.

Trichomoniasis remains a significant public health concern. Globally, the World Health Organization (WHO) reported approximately 156 million new cases in 2016. In India, studies suggest that *T. vaginalis* infection rates range between 8.5–34 % among women attending STI clinics, with higher prevalence in certain regions [2]. For instance, in Gujarat, data indicate a prevalence rate of about 34 % (out of 102 samples, 35 were positive for *T. vaginalis* by the culture method) in women seeking reproductive health services [3]. These numbers underline the importance of efficient diagnostic and therapeutic interventions.

This paper presents a thorough review of the literature and five case studies, comprising four women and one man, diagnosed with trichomoniasis, highlighting the critical role of improved diagnostic practices in disease detection and management.

## Literature review on *T. vaginalis* diagnostic approaches

The detection and management of *T. vaginalis* infections, a leading cause of sexually transmitted infections globally, has seen noteworthy evolution over the decades. As the medical and scientific communities’ understanding of the organism’s behaviour has grown, so too have the methodologies to detect it.

### Traditional diagnostic approaches

#### Direct microscopic examination

Historically, the cornerstone of *T. vaginalis* diagnosis was the direct microscopic examination of vaginal or urethral secretions. This microscopic visualization allowed healthcare professionals to observe the characteristic movement of the motile *T. vaginalis* trophozoites. While the technique offers the advantage of rapid results, its sensitivity largely depends on the freshness of the sample, the expertise of the observer and the concentration of organisms in the sample. Studies have often noted that its sensitivity can be 60–70 % compared to culture-based methods [1].

#### Culture techniques

Culture-based methods soon emerged as a diagnostic tool and were considered the gold standard for many years due to their superior sensitivity. Diamond’s medium became the most used culture system. Culture, while more sensitive than microscopy, comes with its own set of challenges. It is labour-intensive, necessitating specific conditions to maintain the viability of the organism. The longer turnaround time for results (often several days) could delay diagnosis and treatment initiation [4].

### Modern diagnostic techniques

#### Nucleic acid amplification tests (NAATs)

The advent of molecular biology brought about a revolution in *T. vaginalis* detection with the development of NAATs. These tests, including PCR, have become increasingly popular due to their high sensitivity and specificity. They work by amplifying and detecting the genetic material of the organism, making them adept at identifying infections even in asymptomatic carriers. While their accuracy is commendable, these tests are often more expensive, requiring specialized equipment and trained personnel [5].

#### Rapid antigen detection tests

Another notable advancement has been the introduction of rapid antigen detection tests. These immunoassays detect *T. vaginalis* antigens in samples, providing results much more quickly than culture methods. Although their sensitivity does not match that of NAATs, their speed and ease of use make them invaluable in settings where rapid diagnosis is crucial, such as STI clinics [6].

#### Comparative analysis of techniques

Microscopy, being immediate, is excellent for on-the-spot decisions. However, its reduced sensitivity, especially if samples are not fresh or in low-parasite-load scenarios, can be a limitation. Culture, though sensitive, can be too slow for acute care situations. NAATs represent the pinnacle of accuracy but come with increased costs, making them less accessible in resource-constrained settings. Rapid tests, while not as sensitive as NAATs, provide a balanced solution in many clinical scenarios, offering relatively quick results with decent accuracy.

## Current best practices

The optimal diagnostic approach for *T. vaginalis* often depends on multiple factors: the clinical setting, available resources, the urgency of diagnosis and the patient’s symptoms. Many guidelines now advocate for a combination of methods, using rapid tests or microscopy for immediate patient management while employing NAATs for screening programmes, especially in populations with a high prevalence of asymptomatic carriers.

The journey of *T. vaginalis* diagnosis reflects the broader evolution of medical science, where traditional methods give way to or are complemented by advanced techniques, all with the shared goal of enhancing patient care. While each method has its merits, the continued exploration of even more accurate, cost-effective and rapid diagnostic tools will always be a priority in the field.

## Case presentation

### Case 1

A 28-year-old female presented with complaints of vaginal itching and a malodorous, frothy discharge for the past 5 days. The patient reported no significant medical history and denied any previous STIs. Vaginal examination revealed erythema and inflammation of the vaginal walls. Wet mount microscopy confirmed the presence of motile *T. vaginalis* trophozoites, thus confirming the diagnosis of trichomoniasis. The patient was prescribed oral metronidazole 500 mg twice daily, and her symptoms resolved after 7 days of treatment.

### Case 2

A 35-year-old male presented with urethral discharge and dysuria. The patient reported multiple sexual partners and inconsistent condom use. Urethral swab microscopy revealed motile *T. vaginalis* trophozoites, indicating trichomoniasis. The patient received a 2 g single dose of oral metronidazole, leading to the resolution of symptoms within a week.

### Case 3

A 25-year-old pregnant female in her second trimester was screened for STIs as part of routine antenatal care. Vaginal swab microscopy confirmed the presence of *T. vaginalis* trophozoites. The patient was treated with 1 % topical metronidazole gel once daily. Follow-up examinations revealed complete resolution of the infection.

### Case 4

A 22-year-old woman reported lower abdominal pain, pain during intercourse (dyspareunia), white vaginal discharge and bleeding after intercourse. Microscopy revealed motile *T. vaginalis* trophozoites, with a vaginal pH of 7. The patient was prescribed oral metronidazole 500 mg twice daily for 7 days.

### Case 5

A 46-year-old postmenopausal woman with a history of hysterectomy and asthma presented with itching and burning around the perineum, right flank pain. Her vaginal discharge pH was 6, with sluggishly motile *T. vaginalis* trophozoites identified. The patient received oral metronidazole 500 mg twice daily for a week.

Cases 4 and 5 were lost to follow-up so the clinical outcome for these patients is unknown.

## Discussion

### Clinical presentation and diagnostic approaches

The patients in the case series presented with diverse clinical manifestations that spanned from heavy bleeding and white vaginal discharge to lower abdominal pain, dyspareunia and post-coital bleeding. Such diversity of symptomatology is frequently noted in trichomoniasis cases, as supported by the existing literature [4, 6]. It is this wide range of manifestations that makes trichomoniasis a clinical chameleon, often leading to misdiagnosis or underdiagnosis. Therefore, ensuring the accuracy of laboratory diagnosis is of paramount importance for the successful management of each patient’s condition.

#### Protocols to trigger testing for *T. vaginalis*

Given the protean clinical manifestations of *T. vaginalis*, establishing a definitive protocol for testing is imperative. Our guidelines for triggering a diagnostic test for *T. vaginalis* encompass a combination of clinical presentations, risk assessments and patient-specific factors.

- **Clinical presentation.** While asymptomatic cases remain a challenge, commonly encountered symptoms such as vaginal or urethral discharge, itching, pain during intercourse, or post-coital bleeding immediately warrant testing.
- **Risk assessment.** Patients with a history of multiple sexual partners, inconsistent condom use, or known contact with an infected individual are considered high-risk and are thus recommended for testing.
- **Patient-specific factors.** Pregnant women are routinely tested due to the potential complications *T. vaginalis* can pose during pregnancy. Additionally, individuals with a history of recurrent STIs or those presenting with other STI symptoms are also prioritized for testing to rule out co-infections or a misdiagnosed recurrent infection.
- **Other triggers.** Given its ‘clinical chameleon’ nature, any unexplained or persistent reproductive tract symptoms, especially if other tests come back negative, serve as a trigger for *T. vaginalis* testing as a diagnostic precaution.

By adhering to these protocols, we aim to capture a broad spectrum of potential *T. vaginalis* infections, ensuring that even subtle presentations do not go undetected.

Historically, the laboratory diagnosis of *T. vaginalis* has been challenging, primarily due to our inability to cultivate the organism and the transient presence of the protozoa in clinical samples. However, microscopic examination of a wet mount is an effective and rapid tool for diagnosing trichomoniasis. It provides direct visualization of the motile trophozoites of *T. vaginalis*, enabling a swift diagnosis [7]. Additionally, the Gram staining technique, a mainstay in microbiology, provides robust diagnostic support, especially in resource-limited settings where advanced diagnostic tools are not readily available.

Importantly, an accurate diagnosis hinges on proper sample collection. Poor collection methods can yield false negatives, leading to missed or delayed diagnosis and treatment. In this series, correct sample collection has been shown to be pivotal in obtaining accurate diagnoses, revealing an opportunity to improve diagnostic protocols.

#### Shift in diagnostic protocols[8]

Traditionally, our primary diagnostic approach for *T. vaginalis* revolved around culture-based methods, which, while sensitive, often required prolonged turnaround times and specific conditions for organism viability. Recognizing the need for more immediate results, particularly in acute care scenarios, we transitioned to emphasizing wet mount microscopy. This method, involving the direct microscopic visualization of samples, offers rapid results and a practical solution for on-the-spot clinical decisions. While still retaining culture methods for specific scenarios where they might offer added value, the shift towards wet mount microscopy represents our strategic adaptation to the evolving demands of clinical care, balancing speed with diagnostic accuracy.

#### Refining sample collection protocols for optimal diagnosis

Accurate diagnosis of *T. vaginalis* often hinges on the quality of the sample collected. Historically, our sample collection was more generalized and lacked specificity, sometimes leading to compromised sample integrity, especially if not processed immediately. This led to occasional false negatives or inconclusive results. Recognizing this limitation, we introduced rigorous guidelines and training for healthcare personnel involved in sample collection.

#### The new standard emphasizes the following.

- **Timely processing.** Ensuring that samples are transported to the laboratory and processed as swiftly as possible to maintain organism viability.
  To address timely transportation and processing of samples, our facility implemented a decentralized approach. The microbiology laboratory is situated in close proximity to the outpatient services of the hospital and obstetrics and gynaecology outpatient services are among them. This reduced transit time ensured immediate processing, maintaining organism viability. This setup also alleviated the burden on the main laboratory, allowing for more focused and efficient handling of genitourinary (GU) samples.
- **Correct sampling site.** Depending on the patient’s symptoms and gender, emphasizing the correct anatomical site for sample collection.
- **Avoiding contamination.** Ensuring that samples are free from contaminants, including lubricants or douches, which can interfere with microscopy.
- **Volume and technique.** Adequate sample volume and correct swabbing technique to ensure a representative sample.
  For ensuring adequate sample volume with swabs, our team focused on training healthcare professionals in proper swabbing techniques. This included the correct insertion depth, duration of contact with the mucosal surface and rotation of the swab to ensure sufficient sample collection.

By adhering to these refined sampling standards, we aim to enhance the accuracy of the diagnostic process, ensuring that every sample has the highest potential for yielding a true and representative result.

#### Co-testing protocols and outcomes

Recognizing the potential for co-infections and the overlapping symptomatology of various STIs with *T. vaginalis*, we employed a multi-test diagnostic approach in all presented cases, as follows.

- **Microscopy for clue cells.** To rule out bacterial vaginosis, microscopic examinations were conducted to detect the presence of clue cells. None of the female patients exhibited clue cells on their wet mount microscopy.
- **Syphilis testing.** Given the risk and implications of untreated syphilis, all patients underwent a rapid plasma reagin (RPR) test. All tests returned non-reactive, indicating no active syphilis infections among the patients.
- **HIV testing.** Considering the enhanced risk of HIV transmission associated with STIs, HIV tests were also conducted for each patient. All tested negative.

While our facility does not offer chlamydia and gonorrhoea NAATs, we emphasize clinical evaluation and other available tests to achieve a comprehensive diagnostic picture. This thorough approach ensures an understanding of the broader STI status of the patient, paving the way for informed treatment decisions.

#### Treatment protocols for *T. vaginalis*

The choice of treatment regimen for *T. vaginalis* at our facility is dictated by several factors, including the patient’s clinical presentation, co-existing medical conditions and specific circumstances. Below is an overview of our treatment protocols and the rationale for each.

- **Standard treatment.** The primary treatment for uncomplicated *T. vaginalis* infection in non-pregnant individuals is a single dose of oral metronidazole. This approach is backed by its high efficacy, convenience for the patient and compliance assurance.
- **Extended treatment.** In cases where symptoms are more severe or persistent, or where there is a history of recent treatment failures, a 7 day regimen of metronidazole is preferred. This extended duration ensures complete eradication of the infection and minimizes the chances of resistance or recurrence.
- **Pregnant patients.** Guidelines from the Centers for Disease Control and Prevention (CDC) and the World Health Organization (WHO) recommend oral metronidazole as the standard treatment for *T.s vaginalis* in pregnant women [9, 10]. This recommendation is based on its well-established safety profile and the substantial risks associated with *T. vaginalis* infections during pregnancy, which include pre-term delivery and low birth weight. Consequently, topical treatment is no longer advocated as the primary therapy due to its lower efficacy than systemic treatment.
- **Treatment guidelines.** Our protocols align with the treatment recommendations set forth by the WHO and the CDC. Both of these guidelines emphasize tailoring the treatment approach based on the individual’s clinical and demographic factors [9, 10].

By adhering to these standardized protocols and guidelines, we ensure that each patient receives optimal care tailored to their specific needs, ensuring the highest chances of treatment success.

### The role of sensitization and training

Our efforts to improve diagnosis started with a sensitization and training session among healthcare providers. This focused on proper sample collection and reducing unnecessary requests for cultures. As a result, our laboratory observed an improvement in diagnostic practices, leading to better patient management. This outcome aligns with numerous studies that have highlighted the positive impact of similar training interventions on improving clinical–laboratory coordination and thereby enhancing patient outcomes [11].

Such sensitization and training efforts are critical to improving the diagnostic capabilities of health providers. In our cases, not only did these efforts lead to the diagnosis of five new cases of trichomoniasis, but they also contributed to a larger shift in diagnostic strategies, emphasizing the judicious use of diagnostic tools and a deeper understanding of their clinical applications.

#### Training intervention and its transformative impact

To address recurring challenges in sample quality and unnecessary test requisitions, we orchestrated a targeted interactive session with our obstetrics and gynaecology colleagues in May 2023. This initiative was driven by the increasing influx of high vaginal swabs (HVSs) without discernible clinical relevance.

#### Content of the training

Our training was structured around key themes to ensure a comprehensive understanding.

- **Proper sample collection.** Emphasized the critical role of obtaining high-quality samples, discussing the ideal techniques and common pitfalls to avoid.
- **Wet mount microscopy.** Introduced its significance in the rapid and accurate diagnosis of infections like trichomoniasis, illustrating its practical application.
- **Gram staining.** Demonstrated its pivotal role in discerning various infections, particularly in distinguishing between bacterial vaginosis, candidiasis and trichomoniasis.
- **Questioning the necessity of culture.** Given that many causative agents of bacterial vaginosis are anaerobes and both candidiasis and trichomoniasis can be effectively diagnosed with Gram staining and wet mount, we questioned the routine need for culture. This segment emphasized the judicious use of culture, conserving resources and ensuring timely diagnoses.

#### Post-training impact

The results of this training were multifaceted and immensely positive, with most of our cases reporting positive post-training effects.

- **Decrease in unnecessary requests.** There was a marked reduction in superfluous culture and sensitivity requests. This not only conserved resources but also ensured that laboratory efforts were channelled into clinically relevant tests.
- **Enhanced understanding among gynaecologists.** The session armed the obstetrics and gynaecology specialists with insights into proper sample collection and relevant test requisition. Their newfound knowledge ensured that samples sent were of optimal quality and that the requested tests aligned with the patient’s clinical presentation.
- **Collaborative synergy.** This initiative fostered a collaborative environment, bridging the gap between laboratory specialists and clinicians. This synergy promises better patient outcomes, with both teams being well informed and aligned in their approach.

In essence, through this targeted training session, we successfully optimized our diagnostic approach, ensuring efficient utilization of resources and fostering a collaborative spirit between laboratory specialists and clinicians.

### Diagnostic stewardship and its implications

Diagnostic stewardship represents an emerging field of interest, particularly in the face of increasing antimicrobial resistance. This concept emphasizes the need for efficient use of laboratory resources to improve patient outcomes [12]. Through the case series presented, we showcase how proper sample collection and the appropriate use of diagnostic tests, such as wet mount and Gram staining, can provide a reliable and cost-effective solution. Moreover, these practices help reduce unnecessary cultures, which aligns with the global antimicrobial stewardship (AMS) goals, thus contributing to the prevention of antimicrobial resistance [5, 13].

Diagnostic stewardship is increasingly gaining recognition for its potential to improve patient outcomes and reduce healthcare costs. These benefits are not only limited to patients but also extend to the healthcare system as a whole, by optimizing the use of diagnostic tests, preventing unnecessary treatments and reducing the risk of antimicrobial resistance.

Looking ahead, continuous efforts are needed to further promote the principles of diagnostic stewardship and ensure their implementation at all levels of healthcare. By doing so, we can achieve more accurate and timely diagnoses, which will ultimately contribute to improved patient outcomes and greater effectiveness in our battle against antimicrobial resistance.

Through our case series and the subsequent changes in laboratory diagnostic practices, we emphasize the profound impact of correct sample collection, judicious use of laboratory diagnostic tests and effective sensitization and training efforts. Each of these components plays a crucial role in the accurate diagnosis and successful management of trichomoniasis.

### Microscopy within diagnostic stewardship

The ethos of diagnostic stewardship is to ensure that the right test is given to the right patient at the right time. Microscopy, especially wet mount microscopy for the detection of *T. vaginalis*, aligns with these principles in the following ways.

- **Rapid and reliable.** Microscopy provides an immediate visualization of *T. vaginalis* trophozoites, offering swift diagnosis. Its accuracy, when performed correctly, ensures that patients receive timely and appropriate treatment.
- **Cost-effective.** Given the resource constraints in many healthcare settings, microscopy serves as an affordable diagnostic tool. It does not require advanced equipment or reagents, making it accessible and sustainable.
- **Targeted testing.** While our paper may give the impression that microscopy is conducted universally, in practice, it is administered based on clinical judgement. Patients presenting with symptoms consistent with trichomoniasis or those identified as high-risk through our screening protocols are prioritized for microscopic evaluation.
- **Complement to other tests.** In cases where microscopy results are inconclusive or co-infections are suspected, microscopy can act as a stepping stone to more specific tests. It aids in narrowing down differential diagnoses, ensuring that subsequent tests are more focused and are used judiciously.
- **Reduction in unnecessary treatment.** Accurate diagnosis through microscopy reduces the chances of treating non-existent infections. This not only prevents unnecessary drug exposure for patients but also aids in antimicrobial stewardship, reducing the chances of resistance development.

By integrating microscopy within our diagnostic approach, and using it judiciously based on clinical indicators, we align with the principles of diagnostic stewardship. It ensures that our patients receive optimal care, that diagnostics are cost-effective and that resources are utilized efficiently.

### Global implications and the way forward

Our case series underlines the need for strengthening diagnostic capacities, particularly in resource-limited settings where the disease burden of STIs, including trichomoniasis, is often high. It calls for the development and implementation of more such strategic interventions that can empower healthcare providers with the right knowledge and skills to accurately diagnose and manage these infections [14].

Moreover, our findings have broader implications for the global health agenda, specifically for antimicrobial stewardship (AMS). Unnecessary cultures and consequent inappropriate antibiotic use can contribute to the growing menace of antimicrobial resistance. Thus, efficient diagnostic protocols can directly contribute to AMS objectives.

## Conclusion

In conclusion, this case series and literature review underscores the critical role of diagnostic stewardship in the management of *T. vaginalis* infections and other sexually transmitted infections. A paradigm shift towards appropriate diagnostic practices, facilitated by sensitization and training of healthcare providers, can result in more accurate diagnoses, better patient management and significant progress towards global AMS goals.

## References

[1] Schwebke JR, Burgess D (2004). Trichomoniasis. Clin Microbiol Rev.

[2] Madhivanan P, Bartman MT, Pasutti L, Krupp K, Arun A (2009). Prevalence of *Trichomonas vaginalis* infection among young reproductive age women in India: implications for treatment and prevention. Sex Health.

[3] Chakraborty T, Mulla SA, Kosambiya JK, Desai VK (2005). Prevalence of *Trichomonas vaginalis* infection in and around Surat. Indian J Pathol Microbiol.

[4] Sobel JD, Workowski KA, Nyirjesy P (2021). Trichomoniasis in adult women and men. UptoDate.

[5] Baur D, Gladstone BP, Burkert F, Carrara E, Foschi F (2017). Effect of antibiotic stewardship on the incidence of infection and colonisation with antibiotic-resistant bacteria and Clostridium difficile infection: a systematic review and meta-analysis. Lancet Infect Dis.

[6] Kissinger P (2015). *Trichomonas vaginalis*: a review of epidemiologic, clinical and treatment issues. BMC Infect Dis.

[7] Fule SR, Fule RP, Tankhiwale N (2016). A study of wet mount preparations in clinical specimens. Int J Res Med Sci.

[8] Kissinger PJ, Gaydos CA, Seña AC, Scott McClelland R, Soper D (2022). Diagnosis and management of *Trichomonas vaginalis*: summary of evidence reviewed for the 2021 Centers for Disease Control and Prevention Sexually Transmitted Infections Treatment Guidelines. Clin Infect Dis.

[9] (2022). Trichomoniasis - STI Treatment Guidelines [Internet]. https://www.cdc.gov/std/treatment-guidelines/trichomoniasis.htm.

[10] (2024). Trichomoniasis [Internet]. https://www.who.int/news-room/fact-sheets/detail/trichomoniasis.

[11] Luther VP, Shnekendorf R, Abbo LM, Advani S, Armstrong WS (2018). Antimicrobial stewardship training for infectious diseases fellows: program directors identify a curriculum need. Clin Infect Dis.

[12] Curren EJ, Lutgring JD, Kabbani S, Diekema DJ, Gitterman S (2022). Advancing diagnostic stewardship for healthcare-associated infections, antibiotic resistance, and sepsis. Clin Infect Dis.

[13] Abbo LM, Cosgrove SE, Pottinger PS, Pereyra M, Sinkowitz-Cochran R (2013). Medical students’ perceptions and knowledge about antimicrobial stewardship: how are we educating our future prescribers?. Clin Infect Dis.

[14] Gaydos CA, Beqaj S, Schwebke JR, Lebed J, Smith B (2017). Clinical validation of a test for the diagnosis of vaginitis. Obstet Gynecol.

